# Phytochemical, antioxidant and hepatoprotective effects of *Alnus nitida bark* in carbon tetrachloride challenged Sprague Dawley rats

**DOI:** 10.1186/s12906-016-1245-3

**Published:** 2016-08-03

**Authors:** Moniba Sajid, Muhammad Rashid Khan, Naseer Ali Shah, Sayed Afzal Shah, Hammad Ismail, Tahira Younis, Zartash Zahra

**Affiliations:** 1 Department of Biochemistry, Faculty of Biological Sciences, Quaid-i-Azam University, Islamabad, Pakistan; 2 Department of Biosciences, COMSATS Institute of Information Technology, Islamabad, Pakistan; 3 Department of Plant Sciences, Faculty of Biological Sciences, Quaid-i-Azam University, Islamabad, Pakistan; 4 Department of Biochemistry and Molecular Biology, University of Gujrat, 50700 Gujrat, Pakistan

**Keywords:** *Alnus nitida*, Antioxidant, Hepatoprotective, HPLC, Phenolics, Flavonoids

## Abstract

**Background:**

*Alnus nitida* (Spach) Endl. is traditionally used for inflammatory disorders. Diarylheptanoids constituents having diverse therapeutically importance including hepato-protective was reported in *A. nitida*. The aim of this study was to explore the antioxidant and hepato-protective profile of *A. nitida* stem bark’s crude methanol extract (ANM).

**Methods:**

Crude methanol extract of *A. nitida* stem bark and its derived fractions were assessed for phytochemical classes and *in vitro* antioxidant profiling by multidimensional assays. Hepato-protective assessment of ANM was investigated on rats, which were made hepatotoxic using carbon tetrachloride (CCl_4_). Additionally HPLC-DAD analysis of ANM, and its derived ethyl acetate and aqueous fraction was carried out to determine the presence of active constituents.

**Results:**

Qualitative analysis of crude extract-and its fractions depicted the presence of terpenoids, saponins, coumarins, phenols and flavonoids. Maximum quantity of total phenolic content (TPC) and total flavonoid content (TFC) was recorded in ANM and its derived fractions; n-hexane (ANH), chloroform (ANC), ethyl acetate (ANE) and the residual aqueous (ANA). ANM exhibited the best total antioxidant capacity, total reducing power, and scavenging of DPPH and OH radicals. ANE and ANA exhibited strong scavenging potential for iron chelation, nitric oxide and β-carotene bleaching assay. ANM treatment converse the activities of serum-marker enzymes and lipid profile, altered by CCl_4_ treatment in rat. CCl_4_ induced hepatic-cirrhosis in rat resulted in decrease of antioxidant enzyme activities such as catalase, peroxidase, superoxide dismutase, glutathione peroxidase, glutathione-S-transferase and glutathione reductase-which were restored towards the normal level with ANM. Similarly diminished level of reduced glutathione while enhanced level of lipid peroxides, hydrogen peroxide and nitrite in liver of cirrhotic rats was normalized by treatment of ANM. The histopathological studies of liver tissues also represented that ANM possessed the hepato-protective activity. HPLC-DAD analysis against eight known standards confirmed the presence of gallic acid, catechin and rutin in ANM and in ANA while in ANE gallic acid was only detected.

**Conclusion:**

Based on the results of antioxidants, restoration of various antioxidant enzymes and histopathological studies, the recent study concludes that antioxidant potential of *A. nitida* bark might protect the liver damages.

## Background

Plants are currently recognized as an opulent source of antioxidant compounds (e.g., flavonoids, simple phenolics, stilbenes, anthocyanins), which are used chiefly in food industry [1, 2]. It is deliberated that utilization of plant-based antioxidants could be associated with reduced risk of occurrence of numerous human diseases narrated to the oxidative stress [3].

Antioxidants have long been known to protect the biological system through inhibition or deterrence of oxidation stress tempted by reactive oxygen substances produced during usual metabolic activities or environmental factors [4]. Thus, the oxidative stress induced damages of DNA, lipids and proteins may cause various diseases [5].

Liver is an imperious organ in human body [6]. Liver is subordinated with vital functions such as it sustains and normalizes the homeostasis in body. It plays an astounding role in human body as it mediates several biochemical pathways such as body defense against diseases, energy production, and for source of nutrition [7]. Oxidative-stress plays a foremost role in the progress of liver diseases. The liver injury is recruited by the various noxious agents produced by chemicals, viruses or by their bio-activation to chemically reactive metabolites. These metabolites can be free radicals, which either prompts an immune response or directly affects the biochemistry of the cells by cooperating with cellular macromolecules. Even after the encroachment in modern system of medicine, there is deficiency of a trustworthy synthetic liver defensive drug. Hence, natural extracts /products from medicinal plants are considered to be effective for the management of liver disorders [8].

Experimental models of hepatotoxicity can be produced by alcohol, paracetamol, CCl_4_ etc. CCl_4_ is the extreme potent hepatotoxic agent practiced for experimental generation of liver fibrosis [9]. CCl_4_ is metabolized by cytochrome P4502E1 to the trichloromethyl radical (^•^OCCl_3_) and peroxy trichloromethyl radical (^•^OOCCl_3_). It has been reported that one of the cause of CCl_4_-induced liver injury is lipid peroxidation, which is prompted and accelerated by free radical derivatives of CCl_4_ [10].

*Alnus nitida* (spach) Endl belongs to family Betulaceae, ordinarily known as Sharoliin Punjab and Seril iniKashmir, is a deciduousiwoody tree, distributed in westerniHimalayas from Yamuna westwards to Kashmir [11, 12]. *Alnus* species contained diarylheptanoids; a group of natural compounds having 1,7-diphenylheptane skeleton imparting diverse therapeutic effects. About 400 diarylheptanoids [13] have been isolated from different species of *Alnus* showing various pharmacological activities; anti-inflammatory [14, 15], anti-influenza [16], and hepato-protective [17]. Various isolates or the derivatives of diarylheptanoids from *A. japonica* showed antioxidant effects during *in vitro* studies [18, 19]. The diarylheptanoids were also isolated from other medicinal sources such as *Zingiber*, *Curcuma*, *Alpinia* and *Myrica*. Two diarylheptanoids nitidoneiA and nitidone B have been reported from *A. nitida* found in Northern areas of Pakistan [20]. Stem bark of *A. nitida* is pounded and paste is applied externally by the local communities as a remedy for swelling, injuries and pain. Its decoction is also used for internal injuries. Root or stem bark of *A. nitida* is blended with *Urtica dioca, Rumex nepalensis, Zingiber officinale* and paste is used in bone fractures [21]. Extract of *A. japonica* have shown promising effects in alleviating the acetaminophen induced hepatic injury in rat [22]. Hence, the current study was designed to scrutinize the preliminary phytochemical composition, as well as to estimate the antioxidant and hepato-protective activity of methanol extract of *A. nitida* stem bark against CCl_4_ induced hepatotoxicity in rat*.* The crude extract and its derived fractions; ethyl acetate and residual aqueous fractions exhibited significant antioxidant activity were subjected to HPLC-DAD analysis for the presence of polyphenolic constituents.

## Methods

### Reagents and chemicals

Analytical grade chemicals used: sodium carbonate, sodium nitrite, dosium dihydrogen, hydrogen peroxide, ferrous chloride, 2-deoxyribose, potassium ferricyanide, sulphuric acid were bought from Merck. 1,1-diphenyl-2-picryl-hydrazyl, potassium persulphate, 2-ethylbenzothiazoline sulfonic acid, rutin, nitro blue, Folin-Ciocalteu’s reagent, phenazine methosulphate, trichloroacetic-acid and tetrazolium were obtained from Sigma Chemicals Co., St. Louis USA. Oxidized glutathione (GSSG), (DTNB), glutathione (GSH),1,2-dithio-bis-nitroLbenzoic-acid (DTNB), glucose-6-phosphate, thiobarbituric acid (TBA), trichloroacetic acid (TCA), sodium tungstate, perchloric acid (PCA), 2,6-dichlorophenolindophenol, reduced glutathione (GSH), sodium hydroxide, reduced nicotinamide adenine dinucleotide phosphate (NADPH), sodium tungstate, glucose-6-phosphate, rutin, catechin, gallic acid, caffeic acid, apigenin, quercetin, myricetin, and kampferol were bought from Sigma Chemicals Co., USA were used.

### Plant sampling

*Alnus nitida* stem bark was collected from Charbagh town of district Swat, Pakistan during March–April (2015.) The spot of collection was at 34.842727° north latitude and 72.431089° east longitude; at an elevation of 1000 m. Flora of Pakistan [23] was used for the identification of plant and further authenticated by Dr. Sumaira Sahreen, Associate Curator, Pakistan Museum of National History. The authenticated specimen (127963) was held at Pakistan Museum of National History.

### Extract preparation

At room-temperature for 2 weeks the bark of *A. nitida* was air-dried under-shade-and with the help of Willy Mill granulated to 80-mesh size. Bark powder (5 kg) was drenched in-15 L of 95 %-methanol and reprises-the soaking thrice-and filtered the-extract-with WhatmanLNo.L1 filter paper. Rotary evaporator was utilized to dry the filtrate (ANM) under vacuum. Partial purification or separation was done by solvent-solvent extraction in escalating polarity. In the distilled water the extract was suspended and the solvents i.e., n-hexane (ANH), chloroform (ANC) and ethyl acetate (ANE) were used in augmentation order of polarization and as an aqueous extract residual (ANA) was used. Rotary evaporator was used for the evaporation of solvents of all the fractions, and then preserved at 4 °C.

### Phytochemical analysis

Different qualitative tests were employed to identify the phytochemical classes present in the crude methanol extract and various fractions of the stem bark of *A. nitida.*

#### Assessment of phenols

For the presence of phenols previously reported methodology was followed [24]. Each sample (1 mg) was suspended in 2 ml of distilled water containing 10 % ferric chloride. The confirmation sign for the presence of phenol was the development of blue or green color.

#### Assessment of flavonoids

In order to investigate the presence of flavonoids in each sample Trease and Evans protocol [24] was employed. Briefly, 1 mg of every sample was allowed to react with 1 ml of 2 N sodium hydroxide. Appearance of yellow color was considered as the confirmation signal of flavonoid presence.

#### Assessment of coumarins

An amount of 1 mg of each sample was blended with 1 ml of 10 % sodium hydroxide. Appearance of yellow color in the test tube was the evidence of coumarins presence in the sample [24].

#### Assessment of saponins

Each sample (2 mg) was suspended in 2 ml of distilled water and vigorously mixed. The formation of a soapy layer of almost 1–2 cm was the indication of saponins presence [24].

#### Assessment of tannins

The confirmative signal of tannins presence was the development of dim blue or greenish dark shading on the mixing of 1 mg of every sample and 3 ml of 5 % ferric chloride [24].

#### Assessment of terpenoids

Each sample 0.5 mg was mixed with 3 ml of chloroform and 3 ml of concentrated-sulphuric acid. The appearance of red brown colored layer in the middle of two layers confirmed the existence of terpenoids [24].

#### Assessment of anthraquinones

Development of red color was considered as indication for the presence of anthraquinones after mixing of 1 mg of each sample with 2 ml of diluted 2 % hydrochloric acid [24].

#### Assessment of anthocyanins and betacyanins

Each sample (1 mg) was boiled for 10 min in 2 ml of 1 N sodium hydroxide. Formation of bluish green color was the sign of anthocyanin and yellow color formation of betacyanin presence [25].

#### Assessment of alkaloids

An amount of 2 mg of each sample was mixed with concentrated sulphuric acid. The reaction mixture was allowed to react with Mayer’s reagent. Appearance of green color or formation of white precipitates was the symbol of alkaloid presence [25].

### Quantitative analysis

Total phenolic as well as flavonoid contents were quantified by the following narrated procedures.

### Total phenolic contents (TPC)

Spectrophotometric analysis was performed for the analysis of total phenolic content [26]. Each sample (1 mg/ml) was briefly blended with 9 ml of distilled water and 2 ml of Folin Ciocalteu reagent. The acquired mixture was mixed vigorously for 10 min and 10 ml ofL 7 % Na_2_CO_3_ was further mixed in to the mixture. Mixture’s final volume was raised to 25 ml by adding distilled water and then placed into the incubator at room temperature for 60 min. Absorbance of the reaction mixture was measured at wavelength of 750 nm in triplicates for each sample. Gallic-acid was kept as standard, the estimation of TPC was done as mg of gallic acid equivalents (GAE) per gram of dryLextract/fraction.

### Total flavonoid content (TFC)

For the TFC evaluation in the test samples, 0.3 ml of each sample was mixed with 0.25 M NaNO_2_ (0.25 ml) -followed by the addition of 0.1-ml of 0.3 MLAlCl_3_.6H_2_O, and 3.4 ml of-30 % methanol [27]. After 5 min interval 2 ml aliquot of 1 M NaOH was added to it. At the wavelength of 506 nm the absorbance of reaction amalgam was measured against the reagent blank. Content of total flavonoid as-mg rutinLequivalentsLper gram of dryLextract/fraction was assessed by employing the calibration-curve of rutin.

### High performance liquid chromatography (HPLC-DAD) analysis

HPLC analysis of ANM and selected plant fractions (ANE and ANA) was performed using HPLC-DAD (Agilent 1200, Germany) equipped with Zorbex RXC8 (Agilent, USA) analytical column with 5 μm particle size and 25 ml capacity using previously reported method [28]. Each sample was diluted with HPLC grade methanol. Mobile phase was consisted of eluent A, (acetonitrile- methanol--water- acetic acid-/5: 10: 85: 1)-and eluent B (acetonitrile-methanol-acetic acid/40: 60:-1). The gradient (A: B) utilized was the following: 0–20 min (0 to 50 % B), 21–25 min (50 to 100 % B), 26–30 min (100 % B) and 31–40 (100 to 0 % B) at flow rate ofL1 ml/min. The standards and samples were prepared in HPLC grade methanol (1 mg/ml), filtered through 0.45 μm-membrane filter andL20 μl was injected for the analysis. Among the standards rutin was investigated at 257 nm, catechin and gallic acid at 279 nm, caffeic acid and apigenin at 325 nm while quercetin, myricetin and kampferol were analyzed atL368 nm [29]. The analysis was performed in triplicate and the column was reconditioned for 10 min after each run. Quantification was done by the integration of the peak by using the external standard method.

### *In vitro* antioxidant assays

The *in vitro* antioxidant assays were carried out by preparing the plant samples (1 mg/ml) in 95 % methanol and then making its serial dilutions. The specific protocol was followed for finding specific scavenging activities of the plant samples.

#### DPPHL (1, 1-diphenyl-2-picryl-hydrazyl) radical scavenging assay

DPPH scavenging capacity of injurious impacts of free radicals was dictated by following the methodology reported previously [30]. A volume of 100 ml of methanol was used as solvent for 24 mg of DPPH and the stock was kept at 20 °C temperature for further utilization. The dilutions of pre-made DPPH stock solutions were prepared in methanol by optimizing absorbance of DPPH was at 0.908 (±0.02) at wavelength 517 nm. Different concentrations (25–250 μg/ml) of 100 ml of plant samples were mixed with dilution of 3 ml of DPPH. The tubes were thoroughly mixed and placed for 15 min in incubator at room temperature. Absorbance of the reaction mixture was measured at wavelength of 517 nm. Ascorbic acid was utilized as standard to compare the antioxidant activity. Potential as an antioxidant was determined by using Eq. 1:

1 $$ \mathrm{DPPH}\ \mathrm{scavenging}\ \mathrm{activity}\ \left(\%\right)=\frac{\mathrm{Absorbance}\ \mathrm{of}\ \mathrm{control}-\mathrm{Absorbance}\ \mathrm{of}\ \mathrm{sample}}{\mathrm{Absorbance}\ \mathrm{of}\ \mathrm{control}} \times 100 $$

#### Nitric oxide scavenging assay

Nitric oxide scavenging activity of ANM and its derived fractions was estimated by using Griess reagent as the main ingredient [31]. DMSO was used as a solvent for the preparation of plant sample and for serial dilutions. For the development of Griess reagent, equimolar amount of 0.1 % napthylenediamine in distilled water and 1 % of sulphanilamide in 5 % phosphoric acid was added. An aliquot of 0.2 ml of sample was mixed with 0.2 ml of sodium-nitroprusside (10 mM) being formulated in saline phosphate buffer followed by addition of 2 ml aliquot of the Griess reagent to the reaction blend. For 3 h the reaction blend was incubated at room temperature and the absorbance was measured at the wavelength of 546 nm spectrophotometrically utilizing ascorbate as a positive control. For assessing the percentage inhibition of nitric oxide radical formation equation 1 was used.

#### Hydroxyl radical scavenging assay

For measuring the scavenging ability of methanol extract and its fractions of *A.nitida*, Halliwell et al. [32] methodology was followed. DMSO was used as a solvent for the preparation of plant sample and for serial dilutions. As per the procedure, 500 μl of 2.8 mM) 2-deoxyribose was prepared in 50 mM phosphate buffer and pH was maintained at 7.4. The reaction cocktail was made by addition of 0.1 ml of 0.1 M EDTA, 0.2 ml of ferric chloride (100 mM) and 0.2 ml of 200 mM H_2_O_2_ and 0.1 ml of plant sample. To begin the reaction 0.1 ml of ascorbic acid (300 mM) was added and for 1 h placed in incubator at 37 °C. After that 2.9 %-trichloroacetic acid (2 ml) and 1 % w/v thiobarbituric acid (2 ml) prepared in 50 mM NaOH were further added to the reaction cocktail and for 15-min the whole mixture was heated in water bath. The absorbance was measured at wavelength of 532 nm once the mixture temperature falls to room temperature. For the analysis of hydroxyl radical scavenging activity Eq. 2 was applied:

2 $$ \mathrm{Superoxide}\ \mathrm{scavenging}\ \mathrm{activity}\ \left(\%\right)=\left(1-\frac{\mathrm{Absorbance}\ \mathrm{of}\ \mathrm{sample}}{\mathrm{Absorbance}\ \mathrm{of}\ \mathrm{control}}\right) \times 100 $$

#### β-Carotene bleaching assay

The ability of *A. nitida* methanol extract and its fractions for β-carotene bleaching was determined by using the scheme of Dapkevicius et al. [33]. DMSO was used as a solvent for the preparation of plant sample and for serial dilutions. In 10 ml of chloroform 2 mg of β-carotene was added and subsequently added 100 mg of Tween 80Land 20 mg of linoleic acid. Once the chloroform evaporated form the reaction blend, 100 ml of-distilled water was added, and vivaciously vortexed to achieve a uniform emulsion of β-carotene linoleate. In freshly prepared 250 μl of emulsion, 30 μl of plant sample was added and optical density was measured at wavelength of 470 nm at 0 h. Finally the absorbance was measured and recorded after keeping the reaction mixture at 40 °C for 1 h. Catechin served as standard in this assay and % inhibition of β-carotene was determined by Eq. 3:

3 $$ \mathrm{Percentage}\ \mathrm{inhibition}=\frac{\mathrm{Absorbance}\ \mathrm{after}\ 2\ \mathrm{h}}{\mathrm{Initial}\ \mathrm{absorbance}} \times 100 $$

#### Chelating power assay

The iron (II) binding capability at multiple sites confers the antioxidant potential of plant samples [34]. Methanol was used as a solvent for the preparation of plant sample and for serial dilutions. A volume of 200 μl of each dilution was mixed with 900 μl of methanol and 100 μl FeCl_2_.2H_2_O (2.5 mM) and incubated for 5 min. To trigger the reaction 800 μl of ferrozine (6.0 mM) was introduced and afterL15 min of incubation the optical density was recorded at 562 nm using EDTA as standard in comparison. For the evaluation of chelating power following Eq. 4 was employed:

4 $$ \mathrm{Chelating}\ \mathrm{effect}\ \%\kern0.5em =\kern0.75em \left[\mathrm{Absorbance}\ \mathrm{of}\ \mathrm{control}\kern0.5em -\kern0.5em \mathrm{Absorbance}\ \mathrm{of}\ \mathrm{the}\ \mathrm{sample}/\mathrm{Absorbance}\ \mathrm{of}\ \mathrm{control}\right] \times 100 $$

#### Reducing power assay

DMSO was used as a solvent for the preparation of plant sample and for serial dilutions. Briefly, 4 ml of plant extract was taken and mixed with 4 ml of 0.3 M phosphate buffer (pH 6.8) and 4 ml of potassium ferricyanide (10 mg/l) and the reaction cocktail was placed in the incubator for 20 min at 80 °C for 10 min. After addition of 4 ml of trichloroacetic acid (200 mg/l) in the reaction cocktail, 2 ml was of it was diluted with 4 ml of distilled water and 0.6 ml of FeCl_3_ (0.2 %). Optical density of the reaction mixture was taken at 700 nm after 10 min of incubation. Gallic acid was appropriated as a standard [35].

#### Phosphomolybedenum assay

The methodology of Prieto et al. [36] was used to assess the antioxidant capabilities of the plant samples. DMSO was used as a solvent for the preparation of plant sample and for serial dilutions. Accordingly, 0.2 ml of the plant sample was mixed with 2 ml of the reagent solution (prepared by adding 28 mM Na_3_PO_4_ and 0.6 M H_2_SO_4_ with that of 4 mM ammonium molybdate). After incubation at 90 °C in a water bath for 80 min the reaction mixture was cooled down at room temperature and optical density was recorded at 765 nm.

### *In vivo* CCl_4_ induced hepatotoxicity in rats

#### Animals

Six weeks old (180–200 g) Sprague–Dawley-male rats were acquired from the Animal House situated at the Quaid-i-Azam University Islamabad, Pakistan. The animals were maintained at 24 ± 3 °C with a 12 h dark/light cycle at Primate Facility of the Quaid-i-Azam University Islamabad, Pakistan. The animals were bred with basal diet with water ad libitum and were sustained in standard laboratory conditions. The basal diet was composed of 20 % protein (casein), 10 % sucrose, 5 % corn oil, 2 % choline chloride, 1 % vitamin mixture, 3.5 % salt mixture and 5 % fibers (cellulose). The remainder was corn starch up to 100 %. Prior to the research propagation ethical approval (Bch#0275) was obtained from Ethics Committee Quaid-i-Azam University Islamabad. Experiments on animals were performed in accordance with the guidelines of the institute of animal ethical committee, NIH, Islamabad.

### Acute toxicity test

Six week old Male Sprague Dawley rats were kept in fasting conditions for overnight with just water availability. Three animals were intra-gastrically administered with dose-of 50 mg/kg bw and were monitored for mortality rate for 2 weeks. DMSO was used as a solvent for the preparation of extract/fraction samples. No initial progression of toxicity was observed, but the methodology was subsequently followed with augmented amount of doses i.e., 100,-200, 400, 1000, 2000, 3000 and 4000 mg/kg bw of the maximum dose of the extract. Three animals were used for each treatment. Mortality was not noticed at the highest dose of 4000 mg/kg, thus 200 and 400 mg/kg bw doses were selected for the evaluation of hepato-protective propagation activities [37].

#### Experimental design

Eight groups (each having 7 rats) of Sprague–Dawley male-rats were made to contemplate the hepatoprotective effects of ANM. Group I was taken as a control and remained untreated. Olive oil and DMSO (1:1; v/v) at a dose of 1 ml/kg bw was administrated to the Group II orally. Group III was intraperitoneally administered with CCl_4_ (1 ml/kg bw; CCl_4_:Olive oil; 2:8 v/v). An amount of 50 mg/kg bw of silymarin (in DMSO; w/v) as a reference chemical was given to Group IV after 24 h of CCl_4_ treatment. Group V and VI were initially treated with CCl_4_ and then after 24 h, they were orally administered with 200 and 400Lmg/kg bw (in DMSO) of ANM. Animals of Groups VII and VIII received only ANM in DMSO at a dose-of-200 and 400 mg/kg bw, respectively. Once the experiment was accomplished, weight of the animals was recorded. Then the animals were euthanized after ether anesthesia. Blood was collected from heart (atrium), immediately removed the liver and transferred to the chilled saline solution and weighted. One liver portion was prepared for-histopathalogical studies, while the second portion was preserved in liquid nitrogen-and stowed at −80 °C for-added enzymatic and DNA damage investigation.

### Biochemical studies of serum

Different liver marker enzymes were used to perform the liver function tests such as alanine transaminase (ALT), aspartate transaminase (AST), alkaline phosphatase (ALP) and bilirubin in serum was evaluated by standard procedure of AMP Diagnostic kits (Stattogger Strasse 31b 8045 Graz, Austria). Serum level of high density lipoproteins (HDL), low density lipoproteins (LDL), total cholesterol and triglycerides were estimated by following the procedures available on the kits.

### Biochemical studies of liver

Small portion of liver was homogenized by the use of homogenizer. Volume of homogenate was noted and accordingly the 10× homogenate was prepared by mixing with EDTA (1 mM) and phosphate buffer (100 mM). The homogenate was placed for 20 min at 4 °C and then centrifuged at 12,000 × g in order to assemble the supernatant. The protein concentration in theLsupernatant was evaluated-according to the method of Lowry et al. [38] using BSA as standard.

### Catalase assay (CAT)

On the basis of decomposition of hydrogen peroxide CAT actions were analyzed by following the method of Chance and Maehly [39]. In short, 2.5 ml of phosphate buffer (50 mM; pH 6.6) as a solvent for of the supernatant (0.1 ml) and 0.4 ml of H_2_O_2_ (5.9 mM; pH 6.0), change in absorbance was noted for one minute at wavelength of 240 nm in a-spectrophotometer.-A change of 0.01 in-absorbance for one-minute was taken as one unit of CAT activity.

### Peroxidase assay-(POD)

Activities of POD were evaluated based on guaiacol peroxidation [39]. To find the POD activity of supernatant the reaction mixture was prepared by adding 0.3 ml of guaiacol (20 mM), 0.6 ml of H_2_O_2_l(40 mM), and 0.2 ml of the supernatant to 3.5 ml of phosphate buffer (50 mM, pH 5.6) serially. Change in absorbance of the reaction solution at 470 nm was observed for one minute. One unit of POD activity was defined as an absorbance-change-of 0.01 of the-solution-per minute.

### Superoxide dismutase assay-(SOD)

For the assessment of hepatic SOD activity, to the reaction blend after the centrifugation (2500 × g for 20 min-followed by 10,000 × g for 20 min) 300 μl of supernatant along with 1.4 ml of sodium-pyrophosphate buffer (0.053 mM; pH 7.3), 0.2 ml of phenazine-methosulphateL(188 μM) were added to the reaction mixture. To initiate the reaction 0.4 ml of NADH (785 μM) was added and-then stopped after 3 min by adding 2 ml of glacial acetic acid. The color intensity was measured at wavelength of 560 nm [40].

### Glutathione-S-transferase assay (GST)

The principle of this methodology is based on the interaction of GSH andL1-chloro-2,4-dinitrobenzeneL(CDNB)Land the subsequent conjugate made is measured with a spectrophotometer [41]. Shortly, 1.575 ml of phosphate buffer (0.2 M, pH 6.8), 0.3 ml of GSH (2LmM), 0.028 ml of 1-chloro-2,4-dinitrobenzeneL(CDNB; 1LmM) were blended followed by 1.2 ml of supernatant. GST activity was estimated by noting-the change in absorbance at wavelength of 340 nm with a molar extinction-coefficient of 8.6 × 10^3^ M^−1^ cm^−1^.

### Glutathione reductase assay (GSR)

GSR analysis was done by following the method of Carlberg and Mannervik [42]. Supernatant samples (0.2 ml) were amalgamated with 0.1 ml of EDTA (0.5 mM), 1.68 ml of phosphate buffer,-0.04 ml of oxidized glutathione (1 mM), and 0.1 ml of NADPH (0.1 mM). The OD was measured atL340 nm after blending. GST activity was estimated as nM NADPH oxidized/min/mg protein, using a molar extinction coefficient of 8.22 × 10^3^ M^−1^ cm^−1^.

### Glutathione peroxidase-assay (GSH-Px)

The activity of glutathione peroxidase was assayed as described earlier [43]. Hepatic-supernatant (1.2 ml) of every rat was mixed with 1.46 ml phosphate buffer (0.2 M; pH 7.9), 0.1 ml of EDTA (2 mM), 0.2 ml of sodium azide (2 mM), 0.06 ml of glutathione reductase (1 IU/ml), 0.08 ml of GSH (2LmM), 0.2 ml of NADPH (0.4 mM),L0.01 ml of H_2_O_2_ (0.25 mM). The absorbance was noted at 340 nm, and GSH-Px activity was assessed by using a molar extinction coefficient of 6.23 × 10^3^ M^−1^ cm^−1^.

### Reduced glutathione assay (GSH)

The concentration of reduced glutathione was assessed by spectrophotometric technique [44]. The basis of this method-is based on the breakdown-of 1,2-dithio-bis nitro-benzoic acid (DTNB) by sulfosalicylic acid, as a result yellow-color is produced. The yellow color produced was read immediately at 412 nm on a-spectrophotometer and was expressed as μM GSH/g tissue.

### Estimation of lipid-peroxidation assay (TBARS)

The analysis of lipid peroxides as thiobarbituric acid reactive substances (TBARS) was done by following protocol of Iqbal and Wright [45]. Shortly after the ascorbic acid and ferric chloride were mixed with the supernatant it was put in a shaking water bath at 37 °C for 1 h, after that trichloroacetic acid was added. 2.0 ml 0.69 % thiobarbituric acid was added and all the tubes were placed in a water bath to boil for 10 min, and then transferred to crushed ice bath before centrifuging at 2500 × g for 15 min. The quantity of TBARS manufactured in each of the samples was evaluated by measuring the OD of the supernatant atI535 nm against a reagent blank. The outcomes were expressed as nM TBARS/min/mg tissue-at 37 °C using a molar extinction coefficient of 2.58 × M^−1^ cm^−1^.

### Hydrogen peroxide assay (H_2_O_2_)

Oxidation of phenol red in the presence of H2O2 mediated horseradish peroxidase was done to assay hydrogen peroxide (H_2_O_2_) [46]. Horse radish peroxidase (8.5 units), phenol red (0.29 nM), dextrose (6.6 nM) and phosphate buffer (0.04 M; pH 8.0), comprising solution was used for the suspension of homogenate sample (3.0 ml) and incubation was done at 37 °C for 60 min. A volume of 0.01 ml of NaOH (1 N) was mixed following centrifugation at 800 × g for 5 min. Supernatant was checked for absorbance at 615 nm against a reagent blank. H_2_O_2_/min/mg tissue-based concentration was shown on the basis of H_2_O_2_ oxidized phenol red of standard curve.

### Nitrite assay

Equal quantity i.e. 100 μl each of both 5 % ZnSO_4_ and 0.3 M NaOH was used to remove the proteins of tissue samples (100 mg), which were then centrifuged at 6400 *g* for 15–20 min. The supernatant was collected by centrifugation at 6400 × g for 20 min. To the cuvette Griess reagent (1.0 ml) was added, and the spectrophotometer was blanked at wavelength of 540 nm, then the supernatant was added to the cuvette having Griess reagent. Nitrite concentration was computed using a standard curve for sodium nitrite [47].

### Tissue protein estimation

The method of Lowry et al. [38] was used to determine the total amount of soluble protein in tissue homogenate. To the tissue homogenate 200 μl of 1.1 M potassium phosphate buffer-(pH 8.0) was added to dilute the tissue sample. A volume of 1 ml of alkaline copper-solution was added to-this blend, and placed at room temperature. After incubation for 20 min, 200 μl of Folin-Ciocalteu phenol reagent was added. Reaction tubes containing the test mixtures were then vortexed and incubated again atL37°C for 20 min. At 650 nm optical density was measured spectrophotometrically. Total soluble proteins of the tissue samples-were then detected using standard curve of bovine serum-albumin.

### Histopathological studies

Histopathological examination was performed for the hepatic injuries. Samples were paraffin embedded, after fixation in fixative solution consisting of: formaldehyde 20 %, absolute alcohol 70 %, glacial acetic acidL10 %. Sections were made at 4–5 μm, stained with hematoxylin/eosin and were examined under light-microscope-(DIALUX 20 EB).

### Statistical analysis

The parametric data were expressed as the mean- ± SD for the 07 rats in each group. Statistix 8.1 software was used for statistical analysis. After estimating the normality test of all the groups for various parameters the data was analyzed for one way analysis of variance to compute the differences between groups. Post hoc testing was carried out for inter-group comparisons using the least significant difference (LSD) test at *P* > 0.01.

## Results

### Qualitative phytochemical analysis

Table 1 illustrated the phytochemical scrutiny of crude extract and its various fractions. Qualitative analysis ensured the presence of alkaloids, coumarins, tannins, saponins, flavonoids, phenols, betacyanins, terpenoids and anthraquinones in ANM. Terpenoids and anthraquinones were present in ANH. The ANC constituted the various chemicals classes except the alkaloids and betacyanins. Tannins, saponins and betacyanins did not make their presence in ANE. The ANA contained all the chemical classes except the anthraquinones and betacyanins.

**Table 1:** Phytochemical analysis of *A. nitida* bark methanol extract and derived fractions

Compound class	Extract/Fraction				
	ANM	ANH	ANC	ANE	ANA
Alkaloids	+	-	-	+	+
Anthraquinones	+	+	+	+	-
Tannins	+	-	+	-	+
Terpenoids	+	+	+	+	+
Saponins	+	-	+	-	+
Betacyanin	+	-	-	-	-
Coumarins	+	-	+	+	+
Flavonoids	+	-	+	+	+
Phenols	+	-	+	+	+

(+) present, (−) absent

ANM *A*. *nitida* methanol extract, ANH *A*. *nitida* n-hexane fraction, ANC *A*. *nitida* chloroform fraction, ANE *A*. *nitida* ethyl acetate fraction, ANA *A*. *nitida* aqueous fraction

## Plant yield, total phenolics and flavonoid content

The percentage yield of methanol extract and its various fractions are depicted in Table 2. The extraction yield of ANM was 86 g whereas the yield of its fractions; ANH, ANC, ANE and ANA was found to be 7, 24, 21 and 33 g respectively. The equivalents of TPC and TFC were calculated on the basis of regression lines for gallic acids (y = 0.0103x + 0.1875; *R*^*2*^ = 0.9978) and rutin (y = 0.00028x + 0.497; *R*^*2*^ = 0.998) (Table 2). The highest TPC was found in ANM (631.5 ± 1.7 mg GAE/g extract) followed by ANE (607 ± 1.97 mg GAE/g extract), ANA (591.2 ± 1.3 mg GAE/g extract), ANC (527 ± 3.1 mg GAE/g extract). The TFC was found to be maximum in case of ANM (221.5 ± 2.5 mg RE/g extract) followed by ANE (211.2 ± 2.4 mg RE/g extract), ANA (114.8 ± 1.8 mg RE/g extract), ANC (98.6 ± 1.61 mg RE/g extract) (Table 2).

**Table 2:** Estimation of plant extraction yield, total phenolics, flavonoids, antioxidant capacity and reducing power of *Alnus nitida* bark

Sample	Yield (g)*	Total phenolic contents expressed as gallic acid equivalents (mg/g of extract)	Total flavonoid contents expressed as rutin equivalents (mg/g of extract)	Total antioxidant capacity expressed as ascorbic acid equivalents (mg/g of extract)	Total reducing power expressed as ascorbic acid equivalents (mg/g of extract)
ANM	86	631.5 ± 1.7	221.5 ± 2.5	32.6 ± 1.2^a^	125.5 ± 5.0^a,b^
ANH	7	0.00	0.00	3.5 ± 0.5^c^	36.0 ± 2.6^d^
ANC	24	527 ± 3.1	98.6 ± 1.61	7.6 ± 0.9^c^	45.5 ± 3.3^d^
ANE	21	607 ± 1.97	211.2 ± 2.4	19.0 ± 0.6^b^	113.1 ± 2.7^b,c^
ANA	33	591.2 ± 1.3	114.8 ± 1.8	16.5 ± 1.1^b^	102.0 ± 3.3^c^

Values are presented as mean ± SD (*n* = 3). Means with different superscript ^(a–f)^ letters in column are significantly (*P <* 0.01) different from each other. * Yield of ANM is based on the dry powder; the yield of its fractions is based on the yield of ANM

ANM *A. nitida* methanol extract, ANH *A. nitida* n-hexane fraction, ANC *A. nitida* chloroform fraction, ANE *A. nitida* ethyl acetate fraction, ANA *A. nitida* aqueous fraction

### HPLC-DAD analysis of *A. nitida* stem bark

**Fig. 1:**
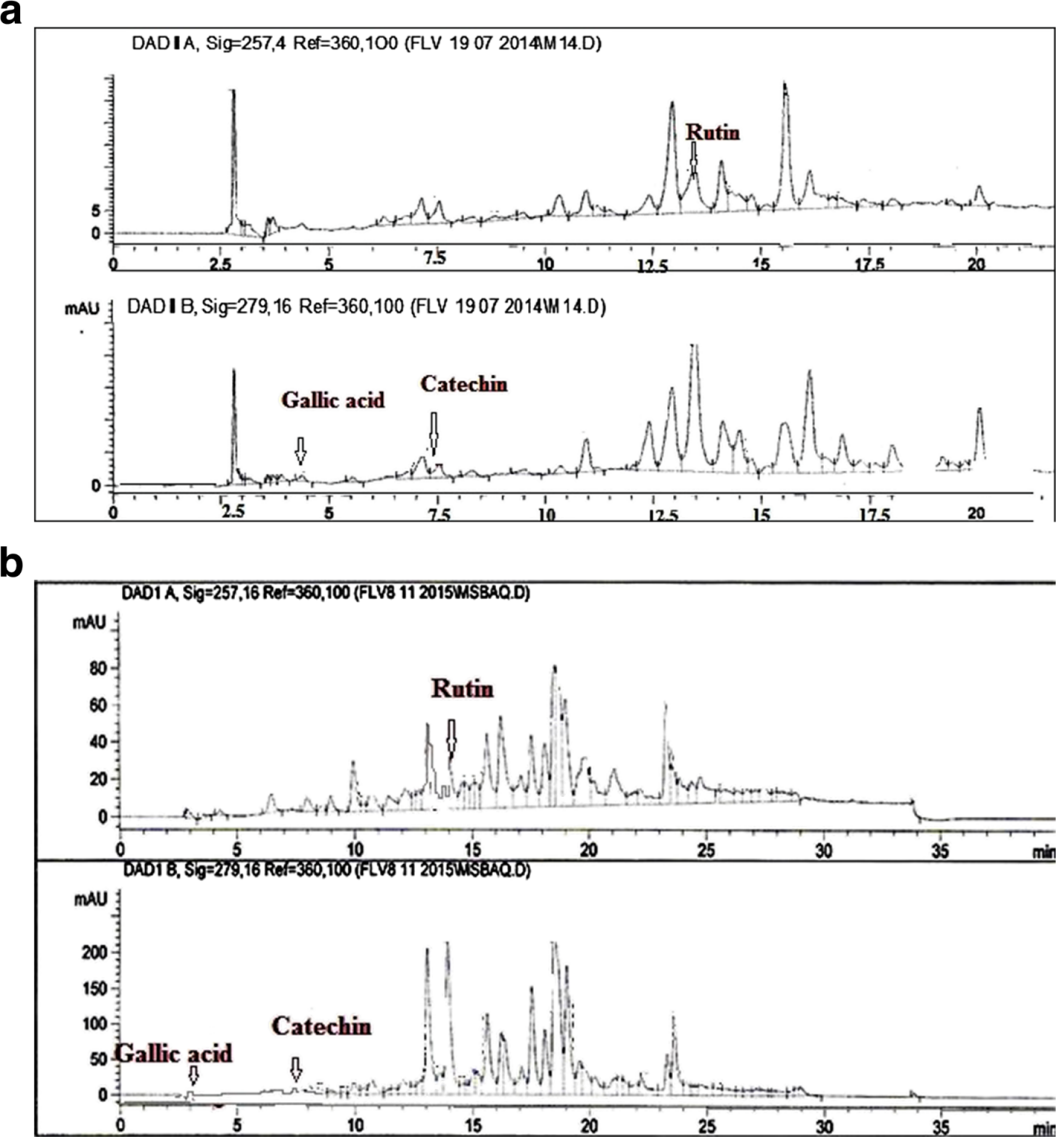
HPLC-DAD chromatogram of ANM (**a**) and ANA (**b**) at different wavelengths Signal 1: 257λ, Signal 2:279λ. Conditions: Mobile Phase A-ACN: MEOH: H_2_O: AA:: 5:10:85:1, Mobile phase B-ACN: MEOH: AA:: 40:60:1, Injection volume 20 μL, Flow rate 1 ml/min, Agilent RP-C8

Reverse phase HPLC analysis was carried out for quantitative estimation of highly potent known antioxidant compounds in our extracts/fractions. In this analysis absorption spectrum and retention times of extracts were compared with reference standards (rutin, catechins, gallic acid, caffeic acid, apigenin, kaempferol, quercetin and myricetin). Results are summarized in Table 3 and a representative chromatogram of ANM and ANA is shown in Fig. 1. From HPLC-DAD profiling of extracts it was observed that the ANA contains gallic acid (550 μg/g), rutin (1659 μg/g) and catechin (2326 μg/g), while ANM contains gallic acid (234 μg/g), rutin (203 μg/g) and catechin (233 μg/g) and ANE contained only the 270 μg/g of gallic acid.

**Table 3:** HPLC-DAD profile of *A. nitida* bark methanol extract, ANE and ANA

Extract	Polyphenolics (μg/g of extract)							
	Rutin	Gallic acid	Catechin	Caffeic acid	Apigenin	Kaempferol	Quercetin	Myricetin
ANM	203	234	233	-	-	-	-	-
ANE	-	270	-	-	-	-	-	-
ANA	1659	550	2326	-	-	-	-	-

ANM *A*. *nitida* methanol extract, ANH *A*. *nitida* n-hexane fraction, ANC *A*. *nitida* chloroform fraction, ANE *A*. *nitida* ethyl acetate fraction, ANA *A*. *nitida* aqueous fraction

### *In vitro* antioxidant assessment

#### DPPH radical scavenging activity

**Fig. 2:**
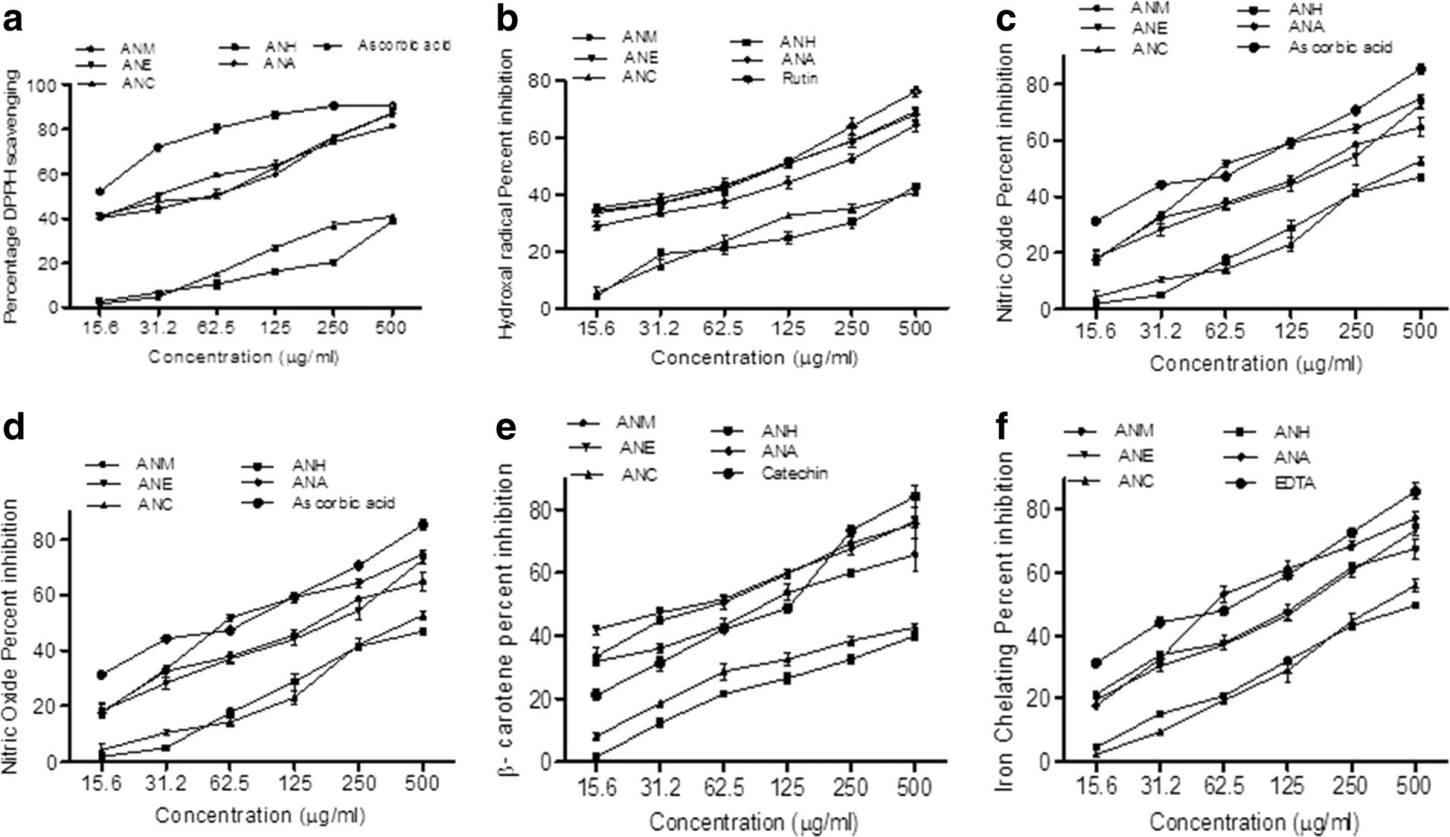
Effect of different concentrations of MBM and its fractions on various *in vitro* antioxidant assays

The scavenging capacity of ANM and its fractions against DPPH are illustrated in Table 4. The quality of antioxidants in the extract and fractions was calculated by the IC_50_ value, low IC_50_ indicates strong antioxidant activity. DPPH radical scavenging effect of various fractions of crude extract was compared with the same doses of ascorbic acid and the outcomes are depicted in Fig. 2a. Minimum IC_50_ values were depicted by ANM (32.0 ± 1.8d μg/ml) followed by ANE (39.5 ± 2.1Iμg/ml) and ANA (43.7 ± 2.9 μg/ml) while ANC and ANH showed the higher IC_50_ values of (633.8 ± 3.9 μg/ml) and (951.5 ± 4.3 μg/ml) respectively. Overall order of IC_50_ of ANM < ANE < ANA < ANC < ANH was observed. The DPPH radical scavenging activity of extract and fractions showed significant correlation with TPC (*R*^*2*^ = 0.86, *P <* 0.05) and with TFC (*R*^*2*^ = 0.91, *P <* 0.01) (Table 5).

**Table 4:** IC_50_ values of different antioxidant activities of ANM and its fractions

	IC_50_ (μg/ml)				
Sample	DPPH scavenging	Hydroxyl scavenging	Nitric oxide scavenging activity	β-carotene bleaching scavenging activity	Iron chelating assay
ANM	32.0 ± 1.8^d^	97.5 ± 2.9^d^	153.6 ± 4.0^c^	103.6 ± 4.2^c^	130.5 ± 4.1^c^
ANH	951.5 ± 4.3^a^	932.5 ± 3.3^a^	465.5 ± 2.8^a^	858.9 ± 3.8^a^	430.2 ± 3.9^a^
ANC	633.8 ± 3.9^b^	812.5 ± 2.9^b^	414.9 ± 2.8^b^	726.2 ± 4.7^b^	347.9 ± 2.2^b^
ANE	39.5 ± 2.1^c,d^	102.7 ± 3.3^d^	151.8 ± 3.1^c^	41.4 ± 1.9^f^	130.1 ± 0.8^c^
ANA	43.7 ± 2.9^c^	173.4 ± 2.0^c^	84.6 ± 3.7^d^	54.7 ± 4.4^e^	76.8 ± 4.5^d^
Rutin	-	83.3 ± 4.2^e^	-	-	-
Ascorbic acid	11.9 ± 0.5^e^	-	56.9 ± 2.5^e^	-	-
EDTA	-	-	-	-	56.0 ± 1.7^e^
Catechin	-	-	-	90.2 ± 2.3^d^	-

Values are presented as mean ± SD (*n* = 3). Means with different superscript ^(a–f)^ letters in the rows are significantly (*P <* 0.01) different from each other

ANM *A*. *nitida* methanol extract, ANH *A*. *nitida* n-hexane fraction, ANC *A*. *nitida* chloroform fraction, ANE *A*. *nitida *ethyl acetate fraction, ANA *A*. *nitida* aqueous fraction

**Table 5:** Correlation of IC_50_ values of different antioxidant activities with total phenolic and total flavonoid contents

Activity	Correlation R^2^	
	TFC	TPC
DPPH scavenging activity	0.91^**^	0.86^*^
Nitric oxide scavenging activity	0.55	0.77^*^
Hydroxyl radical scavenging activity	0.75^*^	0.84^*^
β-carotene bleaching scavenging activity	0.68^*^	0.82^*^
Iron chelating assay	0.85^*^	0.83^*^
Total antioxidant activity	0.79^*^	0.74^*^
Total reducing power Assay	0.80^*^	0.83^*^

*TFC* total flavonoid content, *TPC* total phenolic content

Column with different superscripts are significantly different ^*^, ^**^, indicate *P <* 0.05, *P <* 0.01

### Hydroxyl radical (•OH) scavenging assay

Hydroxyl radical scavenging capacity of an extract is directly linked to its antioxidant capacity. Figure 2b exhibited the radical scavenging ability of plant samples in the following order; ANM < ANE < ANA < ANC < ANH. Lowest IC_50_ values were recorded for crude extract (97.5 ± 2.9 μg/ml) followed by ANE (102.7 ± 3.3Lμg/ml) and ANA (173.4 ± 2.0 μg/ml) while ANC and ANH showed the higher IC_50_ values of (812.5 ± 2.9b μg/ml) and (932.5 ± 3.3 μg/ml) correspondingly. IC_50_ values of ANA, ANC and ANH were remarkably higher from rutin (83.3 ± 4.2 μg/ml). Significant correlation of IC_50_ values of hydroxyl radical scavenging was calculated for TPC (R^2^ = 0.841, *P <* 0.05) as well as for TFC (*R*^*2*^ = 0.753, *P <* 0.05) (Table 4).

### Nitric oxide (NO^−^) scavenging assay

Lowest IC_50_ value of (84.6 ± 3.7 μg/ml) was depicted by ANA comparative to standard ascorbic acid (56.9 ± 2.5 μg/ml). IC_50_ values of other extract/fractions were (151.8 ± 4.0 μg/ml), (153.6 ± 4.0 μg/ml), (414.9 ± 2.8 μg/ml) and (465.5 ± 2.8 μg/ml) for ANE, ANM, ANC and ANH respectively. Figure 2c illustrated the radical scavenging activity of different plant samples relative to standard ascorbic acid. IC_50_ values obtained for nitric oxide scavenging assay exhibited a significant correlation with TPC (*R*^*2*^ = 0.77, *P <* 0.05) and non-significant correlation with TFC (*R*^*2*^ = 0.55, *P* > 0.05) (Table 4).

### Inhibition of β-carotene oxidation

The best results for inhibition of β-carotene oxidation was depicted by ANE and ANA with lowest IC_50_ values of (41.4 ± 1.9 μg/ml) and (54.7 ± 4.4 μg/ml) than that of standard catechin (90.2 ± 2.3 μg/ml). ANM, ANC and ANH showed the IC_50_ values of (103.6 ± 4.2 μg/ml), (726.2 ± 4.7 μg/ml) and (858.9 ± 3.8 μg/ml) respectively. Inhibition of β-carotene oxidation of ANM and its fractions at different concentrations are represented in Fig. 2d. The assay showed significant correlation of IC_50_ with both TPC (*R*^*2*^ = 0.82, *P <* 0.05)-and TFC (*R*^*2*^ = 0.68, *P <* 0.05) (Table 4).

### Iron chelating activity

All the extract/fractions had high levels of ferrous ion chelating activity relative to standard EDTA (56.0 ± 1.7 μg/ml). Figure 2e indicated that the iron chelating activity of plant samples can be ranked as ANA, ANE, ANM, ANC and ANH. The lower IC_50_ values were recorded for ANA (76.8 ± 4.5 μg/ml), ANE (130.1 ± 0.8 μg/ml) and ANM (130.5 ± 0.8 μg/ml). TPC and TFC were significantly correlated (*R*^*2*^ = 0.833, *P <* 0.05)-and (*R*^*2*^ = 0.85, *P <* 0.05) with reducing power respectively (Table 4).

### Reducing power assay

Crude extract (ANM) showed the highest reducing power with 125.5 ± 5.0 mg ascorbic acid equivalent/g followed by ANE (113.1 ± 2.7 mg ascorbic acid equivalents/g sample), ANA (102.0 mg ascorbic acid equivalents/g sample), ANC (45.5 ± 3.3 mg ascorbic acid equivalents/g sample) and ANH (36.0 ± 2.6 mg ascorbic acid equivalents/g sample). There was recorded a significant correlation between the reducing power and with both TPC (*R*^*2*^ = 0.83, P < 0.05) and TFC (*R*^*2*^ = 0.80, P < 0.05) shown in Table 2.

### Total antioxidant capacity (phosphomolybdenum assay)

Total antioxidant capacity of various extracts was estimated by phosphomolybdate method and expressed as equivalents of ascorbic acid-(mg/g of extract). Total antioxidant capacity was found to decrease in the order, ANM > ANE > ANA > ANC > ANH. The assay showed significant correlation with TPC (*R*^*2*^ = 10.74, *P <* 0.05) and TFC (*R*^*2*^ = 0.79, *P <* i0.05) as shown in Table 2.

### Estimation of acute toxicity

The plant crude methanol extract (ANM) and its derived fractions; ANH, ANC, ANE and ANA were found safe at all tested doses (up to 4000 mg/kg) and did not show any noxious symptom in rats like sedation, convulsions, diarrhea and irritation. During the two week assessment, no mortality was found.

### Assessment of biochemical serum markers

The serological concentrations of AST, ALP, ALT and bilirubin are extremely vulnerable to oxidative stress in liver tissues as depicted in Table 6. Chronic CCl_4_ treatment remarkably (*P <* 0.01) boosted the concentrations of serum marker enzymes of liver which was attenuated considerably (*P <* 0.01) by oral administration of ANM. However, ANM at 200 and 400 mg/kg alone exhibited the serum enzyme concentration near to that of control group.

**Table 6:** Effect of different treatments of ANM on liver serum markers profile

Group	ALT (U/l)	AST (U/l)	ALP (U/l)	Bilirubin
Control	45.0 ± 1.56^e^	40.3 ± 1.53^e^	63.7 ± 1.10^d^	0.59 ± 0.02^d,e^
Vehicle control	40.8 ± 1.18^e^	39.4 ± 1.18^e^	60.4 ± 1.13^d^	0.61 ± 0.02^d,e^
CCl_4_ (1 ml/kg)	490.4 ± 5.19^a^	320.9 ± 3.68^a^	298.3 ± 3.29^a^	1.97 ± 0.01^c^
Silymarin + CCl_4_	96.7 ± 2.47^d^	96.9 ± 2.31^d^	108.2 ± 3.98^c^	0.73 ± 0.01^c^
ANM (200 mg/kg) + CCl_4_	137.2 ± 2.13^b^	166.8 ± 2.06^b^	196.7 ± 3.68^b^	1.02 ± 0.10^b^
ANM (400 mg/kg) + CCl_4_	101.4 ± 1.74^c^	121.4 ± 2.74^c^	168.6 ± 2.61^c^	0.66 ± 0.08^c,d^
ANM (200 mg/kg)	52.0 ± 3.99^c,d^	51.7 ± 2.81^d^	73.9 ± 2.75^c^	0.60 ± 0.03^d,e^
ANM (400 mg/kg)	41.24 ± 2.01^e^	37.2 ± 1.01^e^	62.2 ± 2.70^d^	0.59 ± 0.01^e^

Values expressed as means ± SD. Means ± SD with different superscript letter ^(a–e)^ within the column indicate significant difference (*P <* 0.01)

### Assessment of lipid profile of hepatic injury

The protective effect of ANM on the lipid profile such as triglycerides, total cholesterol, HDL and LDL is given in Table 7. The level of triglycerides, total cholesterol, HDL and LDL markedly elevated (*P <* 0.01) in serum of rats after CCl_4_ administration. However, administration of ANM noticeably (*P <* 0.01) decreased the level of these serum markers of lipid profile, dose dependently.

**Table 7:** Effect of different treatments of ANM on lipid profile

Group	Triglycerides (mg/dl)	Total cholesterol (mg/dl)	HDL (mg/dl)	LDL (mg/dl)
Control	83.6 ± 2.64^e^	99.4 ± 2.22^f^	46.1 ± 1.66^e^	32.1 ± 1.28^d,e^
Vehicle control	89.5 ± 2.19^e^	90.3 ± 2.48^f^	43.14 ± 1.70^e^	30.4 ± 1.39^e^
CCl_4_ (1 ml/kg)	372.0 ± 5.63^a^	478.4 ± 5.96^a^	19.2 ± 1.02^a^	208.9 ± 2.89^a^
Silymarin + CCl_4_	120.8 ± 2.44^d^	162.0 ± 2.26^d^	40.0 ± 3.00^e^	43.4 ± 1.55^c^
ANM (200 mg/kg) + CCl_4_	132.8 ± 2.63^b^	187.8 ± 2.63^b^	39.3 ± 2.28^c^	44.1 ± 2.02^b^
ANM (400 mg/kg) + CCl_4_	124.1 ± 2.54^c^	152.1 ± 2.54^c^	41.19 ± 3.96^d^	40.02 ± 1.8^b^
ANM (200 mg/kg)	99.0 ± 3.98^c,d^	92.3 ± 2.98^e^	43.0 ± 2.98^e^	38.0 ± 1.98^c^
ANM (400 mg/kg)	86.7 ± 1.43^e^	87.2 ± 2.42^f^	47.4 ± 1.15^f^	35.1 ± 1.38^d^

Values expressed as means ± SD. Means ± SD with different superscript letter ^(a–f)^ within the column indicate significant difference (*P <* 0.01)

### Assessment of hepatic protein, TBARS,IH_2_O_2_,Initrite and GSH content

The Table 8 showed profile of proteins, TBARS, H_2_O_2_ and nitrite content of different groups. Hepatic protein contents and GSH were reduced by CCl_4_ treatment (*P <* 0.01) in comparison to the control group. Animals administered with ANM (400 mg/kg) alone showed an increase in protein contents and GSH as compared to the ANM (200 mg/kg) + CCl_4_, ANM (400 mg/kg) + CCl_4_ and only CCl_4_ group. CCl_4_ increased the TBARS, H_2_O_2_ and nitrite contents ofJliver homogenatesJas compared to control group (Table 8). Co-administration of ANM comprehensively prevented the rise in TBARS level in a concentration dependent fashion and non-significant difference was observed at higher dose in comparison to that of the control group. Level of H_2_O_2_ and nitrite content was dropped markedly and at the higher dose of ANM their level was statistically similar to that of the silymarin treated group. However, even at the higher dose of ANM their level was significantly higher against the control group. ANM, when treated in the absence of CCl_4_, non-significant alteration in the level of H_2_O_2_ and nitrite content was recorded.

**Table 8:** Effect of different treatments of *A. nitida* stem bark crude extract on liver tissue protein, TBARS, H_2_O_2_, nitrite and GSH content

Group	Protein (μg/mg tissue)	TBARS (nM/min/mg)	H_2_O_2_ (nM/min/mg tissue)	Nitrite content ( μM/ml)	GSH (μM/g tissue)
Control	6.41 ± 0.23^a^	1.52 ± 0.08^d^	6.38 ± 0.15^g^	61.5 ± 1.78^d^	47.0 ± 2.84^a^
Vehicle control	6.14 ± 0.20^a^	1.73 ± 0.07^c,d^	6.77 ± 0.16^g^	64.2 ± 2.83^d^	45.65 ± 2.2^a^
CCl_4_ (1 ml/kg)	3.98 ± 0.16^e^	5.32 ± 0.06^a^	18.3 ± 0.26^a^	106.3 ± 4.67^a^	12.6 ± 1.23^d^
Silymarin + CCl_4_	5.16 ± 0.19^b^	2.11 ± 0.08^b^	8.37 ± 0.15^e^	70.4 ± 0.27^c^	46.7 ± 1.77^a^
ANM (200 mg/kg) + CCl_4_	4.42 ± 0.14^d^	2.11 ± 0.06^b^	12.2 ± 0.21^b^	90.2 ± 2.26^b^	30.74 ± 1.95^c^
ANM (400 mg/kg) + CCl_4_	5.02 ± 0.14^c^	2.02 ± 0.06^b,c^	11.1 ± 0.14^c^	71.1 ± 1.27^c^	41.3 ± 1.68^b^
ANM (200 mg/kg)	5.90 ± 0.16^b,c^	1.81 ± 0.39^b^	7.92 ± 0.29^d^	61.9 ± 2.75^c^	45.4 ± 1.48^a,b^
ANM (400 mg/kg)	6.48 ± 0.32^a^	1.51 ± 0.16^d^	7.50 ± 0.29^f^	60.0 ± 2.45^d^	49.1 ± 1.84^a^

Values expressed as means ± SD. Means ± SD with different superscript letter ^(a–g)^ within the column indicate significant difference (*P <* 0.01)

### Assessment of crude ANM by antioxidant enzymes

Antioxidant polyphenolic compounds have a crucial role in the reclamation of reactive oxygen species-(ROS) and help to sustain cellular balance. In order to characterize the protective effect of ANM, alteration in antioxidant enzyme level was evaluated after CCl_4_ treatment. The Table 9 demonstrates the protective effect of ANM on first phase antioxidant enzymes of liver i.e., CAT, POD and SOD. In comparison to the control group, the level of CAT, POD and SOD in liver tissue was markedly (*P <* 0.01) decreased after the CCl_4_ administration. ANM administration restored the level of these enzymes and at high dose it was statistically similar to that of the silymarin treated group. Oral administration of the ANM alone, illustrated non-significant difference in the activity level of these antioxidant enzymes in comparison to control values.

**Table 9:** Effect of different treatments of *A. nitida* stem bark crude extract on liver tissues antioxidant enzymes

Group	CAT (U/min)	POD (U/min)	SOD (U/mg protein)	GST (nM/min/mg protein)	GPx (nM/min/mg protein)	GR (nM/min/mg protein)
Control	6.68 ± 0.10^a^	10.39 ± 0.22^a^	5.54 ± 0.36^a^	139.8 ± 3.22^a^	92.8 ± 2.05^a^	137.2 ± 2.51^a^
Vehicle control	6.35 ± 0.39^a^	10.06 ± 0.43^a^	5.05 ± 0.62^a^	139.05 ± 3.50^a^	93.3 ± 1.27^a^	133.1 ± 3.51^b^
CCl_4_ (1 ml/kg)	3.49 ± 0.09^e^	5.82 ± 0.12^e^	2.49 ± 0.29^c^	53.77 ± 1.61^e^	32.9 ± 1.79^f^	61.2 ± 1.33^f^
Silymarin + CCl_4_	5.29 ± 0.11^c^	8.57 ± 0.29^b^	4.65 ± 0.32^ab^	122.5 ± 2.41^b^	82.8 ± 1.74^d^	114.5 ± 2.58^d^
ANM (200 mg/kg) + CCl_4_	4.30 ± 0.15^d^	6.20 ± 0.31^d^	3.67 ± 0.27^b^	82.63 ± 1.61^d^	62.4 ± 1.77^e^	86.0 ± 3.05^e^
ANM (400 mg/kg) + CCl_4_	5.61 ± 0.19b^c^	7.57 ± 0.33^d^	4.61 ± 0.29^a,b^	103.8 ± 2.97^c^	85.9 ± 1.48^c,d^	119.19 ± 2.5^c^
ANM (200 mg/kg)	6.19 ± 0.20^b^	9.99 ± 0.24^c^	4.84 ± 0.36^a,b^	140.0 ± 3.18^b^	90.9 ± 2.82^b,c^	125.1 ± 3.31^b^
ANM (400 mg/kg)	6.53 ± 0.28^a^	10.14 ± 0.21^b^	5.73 ± 0.26^a^	141.3 ± 2.77^a^	91.0 ± 0.91^a,b^	136.2 ± 3.02^a^

Values expressed as means ± SD. Means ± SD with different superscript letter ^(a–f)^ within the column indicate significant difference (*P <* 0.01)

The protective effects of ANM on GST, GPx and GR in liver tissue are shown in Table 9. Activity level of GST, GPx and GR enzymes was markedly decreased (*P <* 0.01) with CCl_4_ supplementation in liver tissue when compared to that of the control group. CCl_4_ toxicity was prevented noticeably with co-treatment of ANM in dose dependent manner. Concentration of GPx was found statistically similar to that of the control group at high dose of 400 mg/kg b.w. whereas concentration of GST and GR showed a significant difference. Activity level of GST, GP_X_ and GR at maximum dose of ANM was statistically similar to the silymarin treated group.

### Histopathological examination

**Fig. 3:**
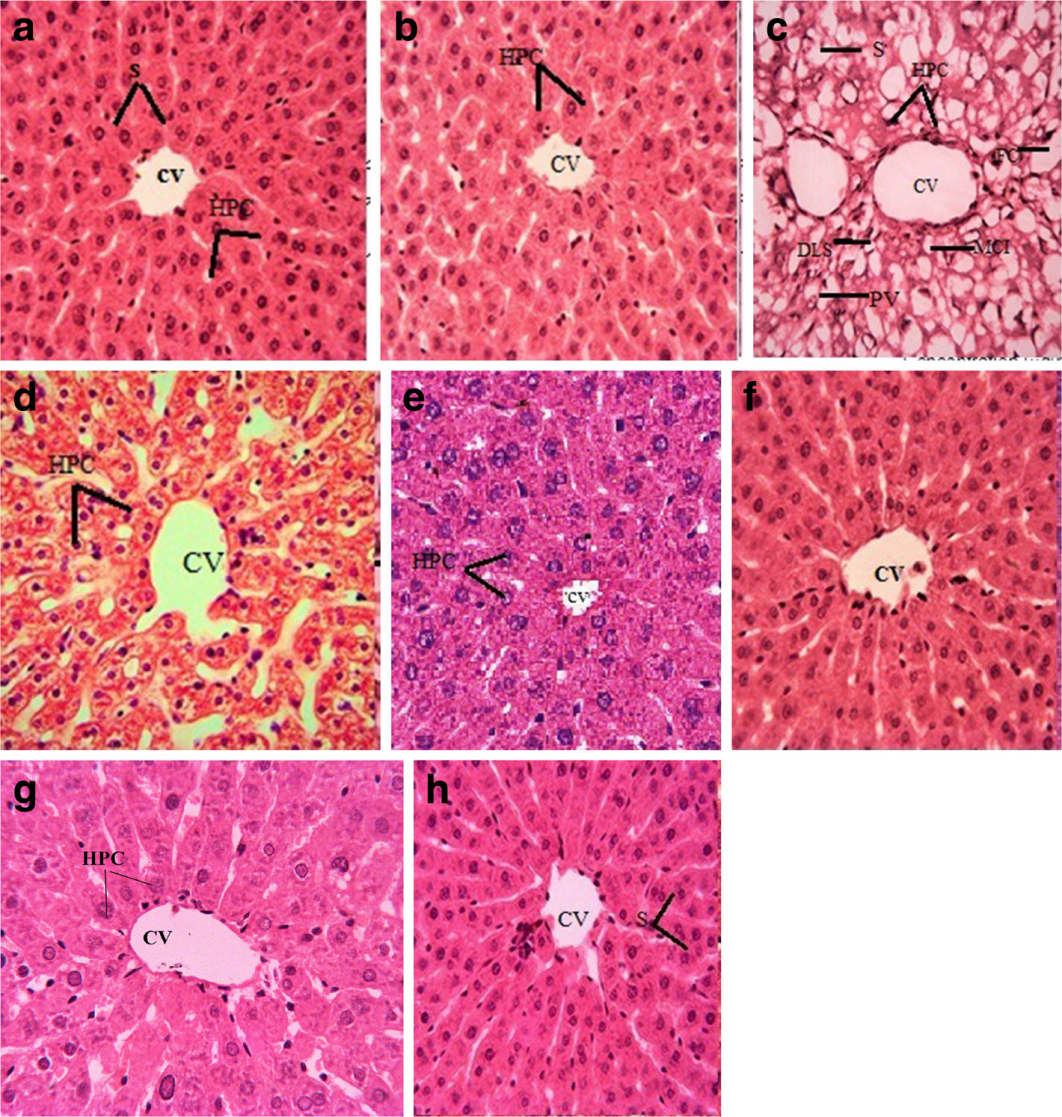
Protective outcome of ANM on histology of hepatic tissues (40× magnification). **a** Control group **b** Vehicle group **c** CCl_4_ 1 m/kg bw, i.p., 30 % in olive oil group, **d** CCl_4_ + Silymarin 200 mg/kg bw group, **e** CCl_4_ + Crude extract 200 mg/kg bw group, **f** CCl_4_ + Crude extract 400 mg/kg bw group, **g** Crude extract 200 mg/kg bw group, **h** Crude extract 400 mg/kg bw group. CV; Central venule, PV; portal vein, HPC; Hepatocytes, S; Sinusoides, DLS; Degeneration of lobular shape, FC; Fatty change, and MCI; Monocyte nuclear cell infiltration

Effect of ANM on liver histology in different groups is illustrated in the Fig. 3. Histology of the liver tissue was performed at 40× to examine morphological alteration as a result of the CCl_4_ treatment and preventive role of ANM against CCl_4_ induced toxicity. Tissue sections were stained with Hematoxylin and Eosin (H & E). In Fig. 3 control group and the negative control group showed the normal architecture of the tissue. Treatment of 1 ml/kg b.w. of 30 % CCl_4_ on alternate days resulted in severe alteration of the histoarchitecture of hepatic tissues. The significant alterations observed in the CCl_4_ treated group were cellular hypertrophy, degeneration of lobular architecture, severe steatosis, congested blood vessels, necrosis, pyknosis, inflammatory cell infiltration, and septa formation. Co-treatment of ANM illustrated protection against toxicity and dose dependent histological protection was observed. At low dose of 200 mg/kg b.w., steatosis, necrosis and inflammatory cells infiltration was prominent whereas at the maximum dose of ANM (400 mg/kg) and in silymarin treated group an appreciable magnitude of protection was exhibited.

## Discussion

Nature has served as opulent repository of medicinal plants for thousands of years and remarkable number of modern drugs has been isolated from natural sources, markedly from plant origin [48]. In the present study, the complete antioxidant profiling of *Alnus nitida* stem bark was carried out *in vitro* and *in vivo* along with HPLC analysis. The preliminary phytochemical analysis of crude extract and its fractions exhibited a wide range of phytoconstituents such as alkaloids, anthraquinones, tannins, terpenoids, saponins, betacyanins, coumarins, flavonoids and phenols. Among the phytochemicals, phenolics and flavonoids have expanded a particular interest because of their wide ranging antioxidant activities. For this purpose polyphenols standards were selected on the basis of their reported medicinal properties for example; catechin and gallic acid have anticancer and antioxidant properties [49], caffeic acid exert the anticancer properties [50]. Rutin has antioxidant, antiviral, antihypertensive and antiplatelet properties [51]. In our analysis gallic acid was detected in crude extract as well as in ANE and ANA fractions. Therefore, it can be concluded that gallic acid is the chief phenolic compound donating to the antioxidant activity of the plant. Additionally, rutin and catechin were also detected in ANA which may be accountable for the increased antioxidant potential of the aqueous fraction. In our results significant positive correlation was found between TPC and TFC which also endorses the HPLC results.

Antioxidants especially phenolic and flavonoid contents have ability to scavenge free radicals such as superoxide and hydroxyl radicals [52]. To investigate the complete antioxidant profile of the *A. nitida* bark five different *in vitro* bioassays (scavenging of DPPH, hydroxyl and nitric oxide, inhibition of β-carotene bleaching and iron chelating) were performed. DPPH radical scavenging is considered as a milestone for assessing the antioxidant potential. In this study ANM, depicted the maximum scavenging followed by ANE and ANA which is expected due to flavonoids which usually contain the high metal chelating activity [53, 54]. *A. nitida* stem bark extract was found to be a powerful scavenger of hydroxyl radicals which are responsible to react with an extensive range of molecules found in living cells [55]. So, scavenging of hydroxyl radical is considered to be a potent antioxidant. In our results, highest activity was showed by the ANM which is coherence with Batool et al. [56] who reported that ethanol extract of *Zanthoxylum alatum* fruit as the most active to scavenge hydroxyl radicals. Nitric oxide has a compelling role in various inflammatory processes and continuous production of this radical is directly toxic to tissues [57]. The results showed that the ANA (IC_50_ 84.6 μg/ml) has highest nitric oxide scavenging activity which is comparable with the ascorbic acid (IC_50_ 56.9 μg/ml). Similar behavior of activity was observed in iron chelating assay which showed the highest activity ANA (IC_50_ 76.8 μg/ml) compared with EDTA (IC_50_ 56.0 μg/ml). As we know that Fe (III) reduction is an indicator of electron donating activity, which is a vital mechanism of phenolic antioxidant action [58] so further study is required to identify the main agent responsible for such strong activity in two different assays. Oxidation of linoleic acid is considered to estimate the antioxidant potency of a plant crude extract and fractions which operates an antioxidant in the reaction mixture to check the consumption of β- carotene by acting on linoleic acid free radicals [59]. The present study depicted the best β-Icarotene scavenging activity of ANE with lowest IC_50_ values even than that of standard catechin attributed to the greater amount of phenolics and flavonoid in ethyl acetate fraction.

Phosphomolybdenum and potassium ferricynide assays are quantitative method to evaluate total antioxidant capacity and total reducing power of the plant extracts respectively. The results of both assays showed similar pattern of contents with the highest values for the ANM followed by its fractions ANE > ANA > ANC > ANH. Our results have been supported by the previous study of Sikder et al. [60] who reported that crude methanol extract of plants exhibited highest value of antioxidant capacity and reducing power respectively. Moreover, all the antioxidant assays showed significant positive correlation with TFC and TPC except DPPH has non-significant correlation with TFC. Collectively, it can be proposed that he antioxidant activity of *A. nitida* may be accredited due to the existence of high content of phenolics and flavonoids which are well recognized as potential antioxidants and free radical scavengers and inhibit lipid peroxidation via the scavenging of radicals and metal chelation.

The antioxidant potential of *A. nitida* can also be described by the fact that the plants of this genus are endowed with terpenoids, flavonoids, diarylheptanoids, phenols, steroids and tannins. Diarylheptanoids are the dominant constituents within the genus *Alnus*, few of them exhibited antioxidant effects and inhibitory effects against nitric oxide and tumor necrosis factor a production [61]. Two diarylheptanoids nitidone A and nitidone B have been reported from *A. nitida* found in Northern areas of Pakistan [20]. About 400 diarylheptanoids [13] have been isolated from different species of *Alnus* showing various pharmacological activities; anti-inflammatory [14, 15], anti-influenza [16], hepatoprotective [17]. Various isolates or the derivatives of diarylheptanoids from *A. japonica* showed antioxidant effects during *in vitro* studies [18, 19].

Liver acts as a center for metabolism of proteins, lipids, carbohydrates, and it also removes unwanted metabolites. Liver secretes a biochemical called bile, which plays an important role in digestion. Based on the significant *in vitro* antioxidant activity, bark of *A. nitida* was evaluated for the hepato-protective effect in CCl_4_ challenged rats. For this purpose, rats were intra-peritoneally administrated with CCl_4_ (1 ml/kg) followed by oral administration of crude extract of *A. nitida* bark with the concentration of 2001 and 400 mg/kg of rats body weight respectively. Administration of CCl_4_ causes hepatopathy which is indicated by elevation in AST, ALT, ALP and bilirubin in serum. Generally, CCl_4_ is metabolized by the liver into highly reactive metabolites which either directly or indirectly cause lipid peroxidation of the hepatocytes [62]. Different cytosolic liver marker enzymes would then leaked out from these swollen and necrotic hepatocytes in to blood circulation and clearly elevated levels are obtained that is related with the immense centrilobular necrosis, ballooning, degeneration and cellular infiltration of the liver [63]. The group administrated with CCl_4_ showed an increase in in serum level of triglycerides, total cholesterol, HDL and LDL (Table 7). The pathological changes in these rats indicated the potential damage in hepatic tissues prompted by CCl_4_ treatment (Fig. 2 and Tables 8 and 9). Our results displayed that treatment of rat with crude extract reduced the toxic effects of CCl_4_ and reestablished the level of above mentioned biochemical similar to untreated group which are supported by previous findings [64]. Moreover, hepatic lesions are also minimized which may be due to presence of flavonoids and phenolics in favorable amount (Fig. 2). Crude extract possibly decreased the oxidative stress by scavenging of free radicals manufactured by CCl_4_ with subsequent renovation in enzymatic activities and oxidative stress indicators; TBARS,IH_2_O_2_, nitrite, protein concentration and GSH in hepatic homogenates. These results are in coherence with Sajid et al. [65] who reported the protective activity of *Artemisia scoparia* against CCl_4_ induced oxidative stress.

Naturally, antioxidant enzymes work in a harmonized fashion to avert the oxidative stress. The metabolic role of liver in detoxification of xenobiotics consequences in the production of ROS, where SOD, CAT, POD, GSH-Px, GST and GSR play a vital role in deterrence of oxidative stress in liver samples [63]. For *in vivo* study, crude extract was selected because of high phenolics and flavonoids content, good antioxidant activity and noteworthy total antioxidant capacity and reducing power. In our study, activities of the antioxidant enzymes in hepatic samples were reduced by CCl_4_ and ameliorated by ANM. Similar protective results have been reported in cirrhotic animals [64].

The hepato-protective proficiencies of *A. nitida* obtained in this study might be also be contributed by other classes of chemicals. Buniatian et al*.* studied hepato-protective properties of altan (obtained on the basis of ellagitannins from the cones of black alder *Alnus glutinosa*) on the model of acute liver damage induced with CCl_4_. It was found that altan exhibits the hepato-protective activity even in a dose of 1 mg/kg which is ten-fold smaller than the dose of traditional flavonoid-based drugs [66]. Antioxidant activity of diarylheptanoids by inhibition of lipid peroxidation has been reported showing the activity of diarylheptanoids more active than α-tocopherol [67]. The anti-hepatotoxic effects of diarylheptanoids and related analogues were assessed utilizing CCl_4_ and galactosamine-induced cytotoxicity in primary cultured rat hepatocytes [68]. So the diarylheptanoids might be the factor along with polyphenols contributing to the antioxidant activity of *A. nitida.*

Histopathological studies of ANM depicted that administration of CCl_4_ prompts extensive fatty change with white and yellow areas due to lipid peroxidation, congestion in blood vessels, cellular hypertrophy, necrotic foci, destruction of the lobular architecture, the development of septa and congested blood vessels with disturbed epithelium and nuclear degeneration in some areas which was significantly recovered by the crude extract (Fig. 2)*.* The study revealed that the crude extract of *A. nitida* bark was comprised of polyphenol and terpenoids which show significant protective effect against hepatotoxicity induced by CCl_4_. Similar histological observation was found by various investigators [69] while assessing the protective effect of medicinal plant againstICCl_4_ and other drugs tempted hepatotoxicity in rats. Taken together our results showed the hepatoprotective effect of *A. nitida* bark against CCl_4_ induced toxicity both by *in vitro* and *in vivo* evaluation techniques.

## Conclusion

Our study concluded that the hepatoprotective activity of *A. nitida* bark is likely due to the scavenging of free radicals and sustaining of endogenous antioxidant molecules. This effect appears to be facilitated by natural antioxidants in *A. nitida* bark, which remarkably diminished the oxidative stress and led to normal hepatic functions. Further exploration must be accompanied to reveal the mechanisms regarding the hepatoprotective effect of *A. nitida* bark at molecular level.

## Abbreviations

ALT, alanine transaminase; ANA, *Alnus nitida* soluble residual aqueous fraction of ANM; ANB, *Alnus nitida* butanol fraction of ANM; ANC, *Alnus nitida* chloroform fraction of ANM; ANE, *Alnus nitida* ethyl acetate fraction of ANM; ANH, *Alnus nitida* hexane fraction of ANM; ANM, *Alnus nitida* methanol extract of bark; AST: aspartate transaminase; BUN, blood urea nitrogen; CAT, catalase; CCl_4_, carbon tetrachloride; GSH, rerduced glutathione; GSR, glutathione reductase; GST, glutathione-S-transferase; POD, peroxidase; SOD, superoxide dismutase; TBARS, thiobarbituric acid reactive substances
